# A deep learning model for predicting multidrug-resistant organism infection in critically ill patients

**DOI:** 10.1186/s40560-023-00695-y

**Published:** 2023-11-09

**Authors:** Yaxi Wang, Gang Wang, Yuxiao Zhao, Cheng Wang, Chen Chen, Yaoyao Ding, Jing Lin, Jingjing You, Silong Gao, Xufeng Pang

**Affiliations:** 1 Department of Critical Care Medicine, The Affiliated Hospital of Qingdao University, No. 16 Jiangsu Road, Qingdao, 266000 China; 2 School of Nursing, Qingdao University, No. 38 Dengzhou Road, Qingdao, 266021 China; 3 Department of Hospital-Acquired Infection Control, The Affiliated Hospital of Qingdao University, No. 16 Jiangsu Road, Qingdao, 266000 China

**Keywords:** Multidrug-resistant organism infection, Backpropagation neural network, Intensive care unit

## Abstract

**Background:**

This study aimed to apply the backpropagation neural network (BPNN) to develop a model for predicting multidrug-resistant organism (MDRO) infection in critically ill patients.

**Methods:**

This study collected patient information admitted to the intensive care unit (ICU) of the Affiliated Hospital of Qingdao University from August 2021 to January 2022. All patients enrolled were divided randomly into a training set (80%) and a test set (20%). The least absolute shrinkage and selection operator and stepwise regression analysis were used to determine the independent risk factors for MDRO infection. A BPNN model was constructed based on these factors. Then, we externally validated this model in patients from May 2022 to July 2022 over the same center. The model performance was evaluated by the calibration curve, the area under the curve (AUC), sensitivity, specificity, and accuracy.

**Results:**

In the primary cohort, 688 patients were enrolled, including 109 (15.84%) MDRO infection patients. Risk factors for MDRO infection, as determined by the primary cohort, included length of hospitalization, length of ICU stay, long-term bed rest, antibiotics use before ICU, acute physiology and chronic health evaluation II, invasive operation before ICU, quantity of antibiotics, chronic lung disease, and hypoproteinemia. There were 238 patients in the validation set, including 31 (13.03%) MDRO infection patients. This BPNN model yielded good calibration. The AUC of the training set, the test set and the validation set were 0.889 (95% CI 0.852–0.925), 0.919 (95% CI 0.856–0.983), and 0.811 (95% CI 0.731–0.891), respectively.

**Conclusions:**

This study confirmed nine independent risk factors for MDRO infection. The BPNN model performed well and was potentially used to predict MDRO infection in ICU patients.

**Supplementary Information:**

The online version contains supplementary material available at 10.1186/s40560-023-00695-y.

## Introduction

Multidrug-resistant organism (MDRO) are bacteria simultaneously resistant to three or more different antibiotics. The infection caused by such bacteria is called MDRO infection [1]. Intensive care unit (ICU) patients are in critical condition and require various invasive procedures [2]. Thus, ICU is deemed the hardest-hit area for MDRO infection in hospitals [3]. Approximately 50% of ICU patients in developing countries suffer from at least one hospital-acquired infection; the corresponding rate for developed countries is 25% [4]. Antibiotic abuse and bacterial mutation have increased the number of MDRO and drug resistance [5]. The World Health Organization stated that bacterial resistance can cause a massive burden of disease, including social expenditure and medical expenditure, and will lead to a decline in global gross domestic product of 1.40–1.60% [6]. The result of a multicenter prospective cohort study showed that the 30-day mortality of patients infected with Carbapenem-resistant *Klebsiella pneumoniae* in China, the United States, and South America were 12.00%, 23.00%, and 28.00%, respectively [7]. However, there is no specific therapy for MDRO infection.

The risk of MDRO infection is early predicted for patients, and appropriate interventions are taken in time, the MDRO colonization rate of ICU patients can be effectively reduced, and reduce the chance of self-infection and cross-infection between patients and healthcare workers [8]. However, the drug sensitivity test and microbial culture results need 24 to 72 h to obtain, resulting in a "lag" in determining the infection status of patients. Given the potential benefits of predictive models in MDRO, many researchers have developed various models based on logistic regression (LR) to predict the risk of MDRO infection [9–11]. Fortunately, LR has the following shortcomings: LR requires a specific linear relationship between the independent and transformed dependent variables. Moreover, the LR model lacked the ability for self-learning and iteration. Once the time and population characteristics changed, the model tended to underperform [12].

The backpropagation neural network (BPNN), one of the most widely used deep learning methods, is a multilayer forward neural network trained according to the error backpropagation algorithm [13]. Compared with traditional LR, the advantage of BPNN is no need for prior knowledge of the mapping relationship between independent and dependent variables. As long as sufficient samples are provided for training, it can complete the nonlinear mapping from input to output variables. BPNN can accept all kinds of independent variables simultaneously without any form of variable transformation, which preserves data information to the greatest extent [14]. In addition, BPNN has strong self-learning and adaptive ability and constantly updates and improves its performance in the use process [12]. The BPNN model has been used to construct disease diagnosis and prognosis prediction models and achieved sound prediction effects [15, 16]. Nevertheless, as far as we know, no study has used it to predict the risk of MDRO infection in ICU patients. Therefore, this study aims to establish the MDRO infection model through BPNN, which identifies high-risk factors and high-risk groups of MDRO infection early and guides the implementation of interventions to reduce the incidence of MDRO infection in ICU patients.

## Methods

### Study population

We retrospectively collected data from patients who received treatment in the ICU of the Affiliated Hospital of Qingdao University from July 2021 to January 2022. The primary cohort enrolled 688 critically ill patients. For external validation, patients in the same study center from May 2022 to July 2022 were selected in the validation set.

All adults (aged ≥ 18 years and ≥ one-time microbial culture performed during ICU hospitalization) in ICU were enrolled in this study. Patients who died or left the ICU within 48 h, had incomplete case data or were diagnosed with MDRO infection prior to ICU admission were excluded. Only the first admission was included for analysis for patients with multiple ICU admissions during hospitalization.

This study has obtained the approval of the Ethics Committee of Qingdao University Medicine (QDU-HEC-2021173). As this study was retrospective and data were anonymized, informed consent was waived.

### Data collection

We obtained patient information through hospital infection surveillance and electronic medical records systems. Initial candidate factors may be associated with MDRO infections, including general data, invasive procedures, medication, laboratory indicators, and the scores. General data included gender, age, body mass index, length of hospitalization, length of ICU stay, and comorbid diseases (including diabetes, hypertension, chronic lung disease, liver disease, chronic renal disease, congestive heart failure, and cerebrovascular disease). Invasive procedures included surgical situations, mechanical ventilation, central venous catheters, gastrointestinal decompression, peripherally inserted central venous catheters, extracorporeal membrane oxygenation, urinary tube, and other drainage tubes in ICU. Medication included antibiotic use, hormone, and nutritional support therapy during ICU. Laboratory indicators included albumin, prealbumin, C-reactive protein, procalcitonin, white blood cells, blood–urea–nitrogen, and creatinine within the first 24 h of their ICU stay. The scores included the APACHE II score, Glasgow coma scale, and nutrition risk screening (NRS)-2002 score within 24 h of admission in the ICU. The diagnosis of the combined disease was as per the International Classification of disease-10 code [17].

This study obtained specimens for microbiologic cultures from blood, urine, sputum, pus, drainage fluid, and secretions. VITEK2 Compact System automatic microbial identification and drug sensitivity analysis system were used for culture identification of strains, and the Kirby Bauer paper diffusion method was applied to the drug sensitivity test of strains. The definition of MDRO was based on the provisional standard definition of MDRO published by Magiorakos and other experts [18]. Long-term bed rest refers to being bedridden for 15 days at least, and more than 90% of the time in bed within 1 day. The surgical situation included the grading of the operation, the classification of incision, and the healing of the incision.

### Screening for risk factors

Patients were categorized into MDRO-infected and non-MDRO-infected groups in accordance with the presence or absence of MDRO infection during the ICU. We combined Lasso and stepwise regression to screen risk factors. Lasso regression used tenfold cross validation to select the optimal penalty coefficient (lambda). The variables whose coefficients were not zero had a significant relationship with the dependent variable and were preserved [19]. Lasso can avoid adding too many independent variables into the BPNN model, thereby reducing the network's complexity and computation and improving the model's prediction accuracy. Then, stepwise regression was applied to further select the optimal combination of independent variables. This method was the introduction of variables one after the other. After introducing a new variable, the old variables that had been selected in the regression model were tested one by one, and the variables that were not meaningful were deleted [20]. This process continued until no new variables were introduced and no old variables were deleted. Variables with bilateral *P* < 0.05 were identified as independent risk factors for MDRO infection.

### Development and validation of the BPNN model

These confirmed independent risk factors for MDRO infection were used as input variables to construct a BPNN model. The BPNN algorithm employed gradient descent to continuously adjust the weights and thresholds among layers through backpropagation to minimize the sum of error squares of the network [21].

These data of the primary cohort were randomly divided into a training set and a test set in an 8:2 ratio, where the training set was utilized to construct the model, and the test set was utilized to evaluate the model's ability to discriminate new samples. To further evaluate the generalization ability and universality of the model, external validation was performed by period validation, that is, patients from the same study center at different times. At this stage, patient data were mainly collected based on independent risk factors confirmed during model construction.

### Statistical analysis

All variables in this study had less than 5% missing values, and mean interpolation was accomplished. Outliers were values that were less than the difference between the first quartile and 1.5 quartile spacing or more than the sum of the third quartile and 1.5 quartile spacing. Outliers in the data were replaced using mean values [22].

Continuous data were described as means ± standard deviation or median and interquartile range (IQR), and group comparisons were performed using the Students' *t* test or Mann–Whitney *U* test. Categorical data were expressed as frequency and percentage, and comparisons were made using the Chi-square or Fisher's exact test between groups.

In this study, Lasso and stepwise regression were performed using "glmnet" and "MASS" packages of R 4.2.3. The BPNN model was constructed with the "nnet" package of R 4.2.3. The model's predictive performance was evaluated in terms of calibration and discrimination. The discrimination was assessed by accuracy, sensitivity, specificity, and area under the curve (AUC). Calibration curves investigated the calibration of the model.

## Results

### Baseline characteristics

**Fig. 1:**
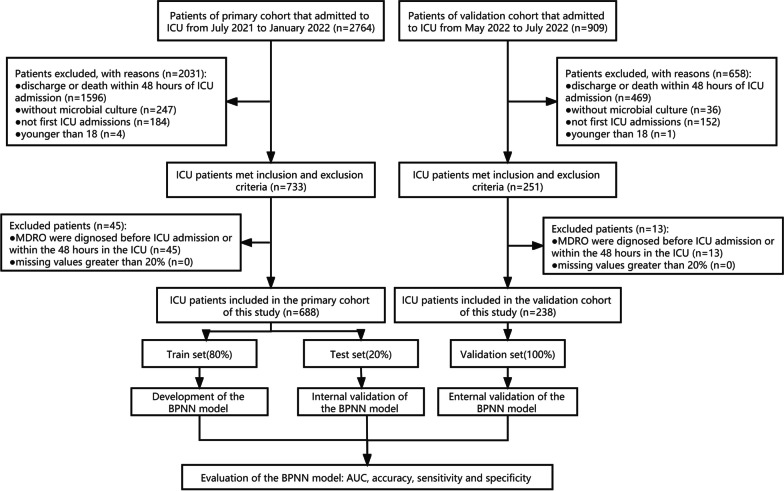
Flowchart for patients selection

Figure 1 shows the flow chart of patient screening. There were 3673 patients enrolled, including 2764 and 909 patients in the primary cohort and validation set. In the primary cohort, 2031 patients were eliminated due to not meeting inclusion and exclusion criteria. Ruling out 46 patients with community-acquired MDRO infection, a total of 688 patients were identified, including 550 patients in the training set and 138 in the test set. The incidence of MDRO infection in the ICU was 15.84% (109/688), with the highest detection rate of Carbapenem-resistant Acinetobacter baumannii (CR-AB). The detection rate of other types of drug-resistant bacteria was depicted in Additional file 1: Table S1. There were 259 (37.65%) females and 429 (62.35%) males. The median age was 65.50 (IQR, 53.00–74.00) years. The body mass index was 23.88 (IQR, 21.48–26.44) kg/m^2^. The length of hospitalization and ICU stay were 19.00 (IQR, 11.00–29.00) days and 9.00 (IQR, 5.00–16.00) days, respectively (Table 1).

**Table 1:** Demographic and clinical characteristics at baseline in the primary cohort

Variables	Total (*n* = 688)	Non-MDRO (*n* = 579)	MDRO (*n* = 109)	*P* value
Age (years)	65.50 (53.00–74.00)	66.00 (52.00–74.00)	65.00 (55.00–74.50)	0.837
Sex, male, *n* (%)	429 (62.35)	360 (62.2)	69 (63.3)	0.824
Length of hospitalization (days)	19.00 (11.00–29.00)	16.00 (10.00–26.00)	32.00 (24.00–52.00)	< 0.001
Length of ICU stay (days)	9.00 (5.00–16.00)	8.00 (5.00–13.00)	19.00 (11.00–30.00)	< 0.001
Long-term bed rest, *n* (%)	361 (52.5)	263 (45.40)	98 (89.90)	< 0.001
Chemoradiotherapy, *n* (%)	36 (5.2)	26 (4.50)	10 (9.20)	0.044
ICU hospitalization history, *n* (%)	139 (20.2)	96 (16.6)	43 (39.4)	< 0.001
Antibiotics use before ICU, *n* (%)	212 (30.8)	141 (24.4)	71 (65.1)	< 0.001
Invasive operation before ICU, *n* (%)	141 (20.5)	87 (15.0)	54 (49.5)	< 0.001
APACHE II score	17.00 (12.00–21.00)	16.00 (12.00–21.00)	21.00 (16.00–26.00)	< 0.001
GCS score	14.00 (10.00–15.00)	15.00 (10.50–15.00)	12.00 (6.00–15.00)	< 0.001
NRS-2002 score	3.00 (3.00–4.00)	3.00 (3.00–4.00)	4.00 (3.00–5.00)	< 0.001
Body Mass Index	23.88 (21.48–26.44)	23.88 (21.30–26.42)	24.22 (21.97–26.66)	0.505
Diabetes, *n* (%)	186 (27.0)	157 (27.1)	29 (26.6)	0.912
Hypertension, *n* (%)	299 (43.5)	242 (41.8)	57 (52.3)	0.043
Hypoproteinemia, *n* (%)	26 (3.8)	13 (2.2)	13 (11.9)	< 0.001
Tumor, *n* (%)	146 (21.2)	124 (21.4)	22 (20.2)	0.773
Cerebrovascular disease, *n* (%)	125 (18.2)	98 (16.9)	27 (24.8)	0.051
Cardiovascular disease, *n* (%)	158 (23.0)	127 (21.9)	31 (28.4)	0.138
Chronic lung disease	85 (12.4)	52 (9.0)	33 (30.3)	< 0.001
Liver disease, *n* (%)	35 (5.1)	28 (4.8)	7 (6.4)	0.489
Renal disease, *n* (%)	53 (7.7)	41 (7.1)	12 (11.0)	0.158
Hematological disease, *n* (%)	14 (2.0)	12 (2.1)	2 (1.8)	1.000
Lymphoma, *n* (%)	2 (0.3)	2 (0.3)	0 (0.0)	1.00
Rheumatic disease, *n* (%)	10 (1.5)	9 (1.6)	1 (0.9)	0.941
Other diseases, *n* (%)	22 (3.2)	19 (3.3)	3 (2.8)	1.000
Albumin (g/L)	31.00(27.78–34.40)	31.10(27.80–34.55)	30.30(27.70–33.85)	0.306
Prealbumin (mg/L)	137.50(89.00–189.23)	136.00 (88.00–188.70)	141.40 (100.95–195.75)	0.295
C-reactive protein (mg/L)	39.11 (9.13–122.64)	39.11 (8.70–125.28)	42.71 (14.43–117.63)	0.503
Procalcitonin (ng/ml)	0.40 (0.11–2.84)	0.41 (0.10–2.83)	0.40 (0.12–2.83)	0.823
White blood cell (10^9^/L)	11.21 (8.00–14.79)	11.33 (7.89–14.80)	10.72 (8.30–14.62)	0.695
Creatinine (μmol/L)	84.05 (63.00–123.10)	83.90 (63.12–122.70)	87.80 (63.00–125.00)	0.67
BUN (mmol/L)	7.60 (5.02–12.16)	7.32 (4.90–11.71)	8.50 (6.28–12.74)	0.045
Number of previous operations	1.00 (0.00–2.00)	1.00 (0.00–1.50)	1.00 (0.00–2.00)	0.385
Mechanical ventilation, *n* (%)	456 (66.3)	367 (63.4)	89 (81.7)	< 0.001
Time for mechanical ventilation (days)	3.00 (0.00–10.00)	3.00 (0.00–8.00)	10.00 (2.00–18.00)	< 0.001
Central venous catheter, *n* (%)	370 (53.8)	305 (52.7)	65 (59.6)	0.181
Time for central venous catheter (days)	2.50 (0.00–7.00)	2.00 (0.00–6.00)	4.00 (0.00–11.00)	0.007
PICC, *n* (%)	72 (10.5)	51 (8.8)	21 (19.3)	0.001
Time for PICC (days)	0.00 (0.00–0.00)	0.00 (0.00–0.00)	0.00 (0.00–0.00)	0.001
ECMO, *n* (%)	13 (1.9)	10 (1.7)	3 (2.8)	0.736
Time for ECMO (days)	0.00 (0.00–0.00)	0.00 (0.00–0.00)	0.00 (0.00–0.00)	0.461
Gastrointestinal decompression, *n* (%)	171 (24.9)	138 (23.8)	33 (30.3)	0.153
Time for gastrointestinal decompression (days)	0.00 (0.00–0.00)	0.00 (0.00–0.00)	0.00 (0.00–3.00)	0.045
Dialysis, *n* (%)	48 (7.0)	35 (6.0)	13 (11.9)	0.027
Time for Dialysis (days)	0.00 (0.00–0.00)	0.00 (0.00–0.00)	0.00 (0.00–3.00)	0.021
Catheter, *n* (%)	631 (91.7)	530 (91.5)	101 (92.7)	0.696
Time for catheter (days)	7.00 (4.00–13.00)	6.00 (3.00–11.00)	12.00 (7.00–18.00)	< 0.001
Number of other tubes	0.00 (0.00–1.00)	0.00 (0.00–1.00)	0.00 (0.00–1.00)	0.013
Time for other tubes (days)	0.00 (0.00–4.25)	0.00 (0.00–4.00)	0.00 (0.00–9.00)	0.001
Hormone, *n* (%)	199 (28.9)	146 (25.2)	53 (48.6)	< 0.001
Enteral nutrition, *n* (%)	402 (58.4)	315 (54.4)	87 (79.8)	< 0.001
Time for enteral nutrition (days)	3.00 (0.00–10.00)	2.00 (0.00–8.00)	10.00 (2.00–17.00)	< 0.001
Parenteral nutrition, *n* (%)	390 (56.7)	328 (56.6)	62 (56.9)	0.964
Time for parenteral nutrition (days)	2.00 (0.00–5.00)	2.00 (0.00–5.00)	3.00 (0.00–8.00)	0.217
Carbapenem antibiotics use, *n* (%)	217 (31.5)	165 (28.5)	52 (47.7)	< 0.001
Third-generation cephalosporin antibiotics use, *n* (%)	131 (19.0)	113 (19.5)	18 (16.5)	0.464
Quantity of antibiotics (categories)	1.00(1.00–1.00)	1.00 (1.00–1.00)	1.00 (1.00–2.00)	< 0.001
Time for using antibiotics (days)	8.00 (4.00–13.00)	7.00 (4.00–12.00)	12.00 (8.00–18.00)	< 0.001
Time for antibiotics combination (days)	0.00 (0.00–3.00)	0.00 (0.00–2.00)	2.00 (0.00–10.00)	< 0.001
Level of operation, *n* (%)				0.418
Level 1	7 (1.0)	6 (1.0)	1 (0.9)	
Level 2	23 (3.3)	20 (3.5)	3 (2.8)	
Level 3	112 (16.3)	94 (16.2)	18 (16.5)	
Level 4	194 (28.2)	171 (29.5)	23 (21.1)	
Classification of incision, *n* (%)				0.103
I	173 (25.1)	150 (25.9)	23 (21.1)	
II	103 (15.0)	93 (16.1)	10 (9.2)	
III	17 (2.5)	13 (2.2)	4 (3.7)	
Healing of the incision, *n* (%)				0.006
A	287 (41.7)	253 (43.70)	34 (31.20)	
B	5 (0.7)	2 (0.30)	3 (2.80)	
C	1 (0.1)	1 (0.20)	0 (0.00)	

Level of operation: Level 1, minimal risk to the patient independent of anesthesia. Level 2, minimal to moderately invasive procedure. Level 3, moderate to significantly invasive procedure. Level 4, highly invasive procedure. Classification of incision: I, an uninfected operative wound. II, clean-contaminated. III, open, fresh, accidental wounds. Healing of the incision: A, inflammatory phase. B, proliferative phase. C, remodeling phase. MDRO: multidrug-resistant organism; ICU: intensive care unit; APACHE II: acute physiology and chronic health evaluation II; GCS: Glasgow coma scale; NRS: nutrition risk screening; BUN: blood–urea–nitrogen; PICC: peripherally inserted central venous catheters; ECMO: extracorporeal membrane oxygenation

Excluding 658 patients without meeting inclusion and exclusion criteria and 13 patients with non-first ICU admission, 238 patients were enrolled to validate externally. The prevalence of nosocomial infection of MDRO was 13.00% (31/288). The specific characteristics of the patients are summarized in Table 2. The comparisons of parameters between the primary cohort and validation set were presented in Additional file 1: Table S2.

**Table 2:** Demographic and clinical characteristics of patients in the validation set

Variables	Total (*n* = 238)	Non-MDRO (*n* = 207)	MDRO (*n* = 31)	*P* value
Length of hospitalization (days)	18.00 (10.00–29.00)	17.00 (9.00–28.00)	26.00 (15.00–45.00)	0.004
Length of ICU stay (days)	8.00 (5.00–14.00)	8.00 (5.00–13.00)	19.00 (11.00–30.00)	0.013
APACHE II score	15.00 (11.00–21.00)	8.00 (5.00–13.00)	11.00 (7.00–17.00)	< 0.001
Quantity of antibiotics (categories)	1.00 (1.00–1.00)	1.00 (1.00–1.00)	1.00 (1.00–2.00)	< 0.001
Long-term bed rest, *n* (%)	62 (26.10)	40 (19.30)	22 (71.00)	< 0.001
Antibiotics use before ICU, *n* (%)	65 (27.30)	46 (22.20)	19 (61.30)	< 0.001
Invasive operation before ICU, *n* (%)	36 (15.10)	19 (9.20)	17 (54.80)	< 0.001
Chronic lung disease	27 (11.30)	20 (9.70)	29 (22.60)	0.070
Hypoproteinemia, *n* (%)	10 (4.20)	7 (3.40)	3 (9.70)	0.250

MDRO: multidrug-resistant organism; ICU: intensive care unit; APACHE II: acute physiology and chronic health evaluation II

### Independent risk factors for MDRO infection

**Fig. 2:**
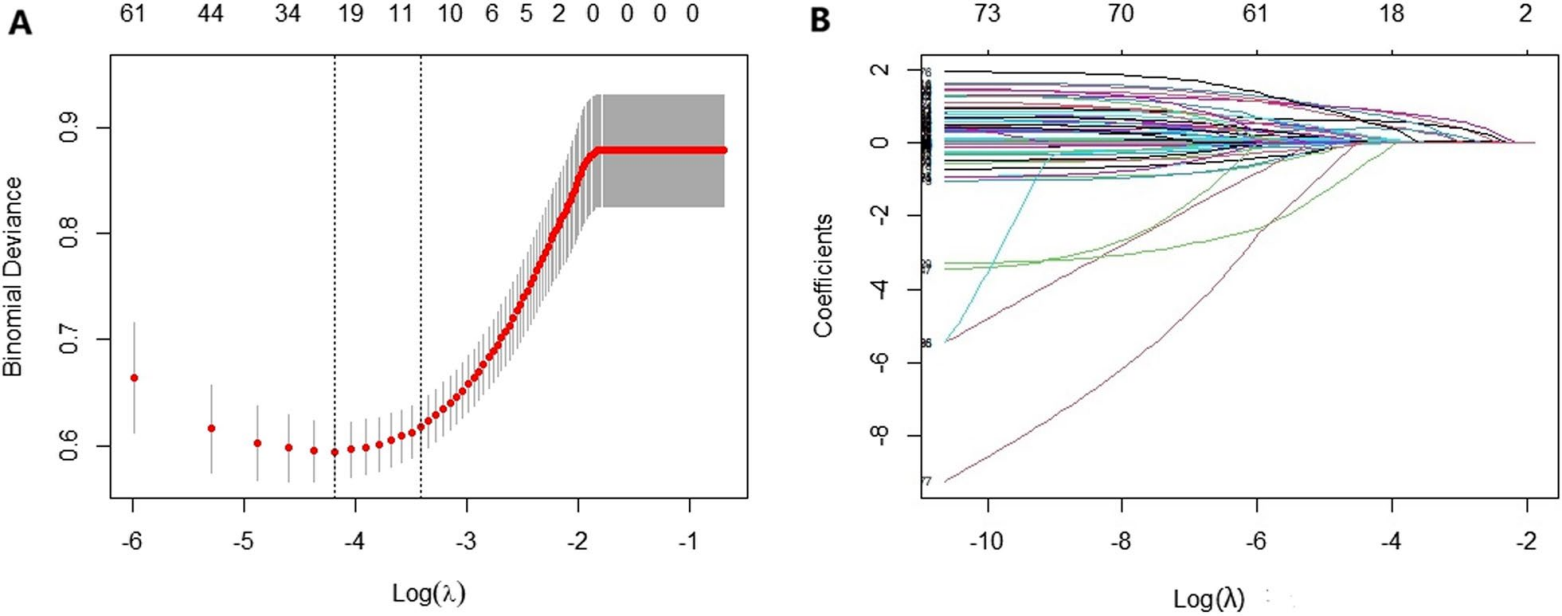
Features selection by Lasso. **A** Tenfold cross validation for the optimal lambda (λ) parameter selection in the LASSO model. There are two dashed lines in the cross-validation diagram, one is the input value with the minimum Mean Square deviation and the other is the input value of the minimum Mean Squared Error(MSE). We take the value of λ with the minimum MSE as the optimal λ. **B** Binomial deviance curve was plotted versus log (λ), where λ is the tuning parameter Lasso regression cross-validation results. LASSO: least absolute shrinkage and selection operator

In our study, lasso adopted nested tenfold cross verification to select the largest lambda with mean error within one standard deviation (lambda.1se) as the optima lambda. As shown in Fig. 2, the optimal lambda was 0.033, corresponding to 11 variables with non-zero coefficients: NRS-2002 score, APACHE II, number of antibiotics and duration of combination, chronic lung disease, hypoproteinemia, invasive operation before ICU, antibiotic use before ICU, length of ICU stay, long-term bed rest.

On this basis, variables were further analyzed using backward stepwise regression. APACHE II (OR 1.06, CI 1.02–1.10; *P* = 0.002), quantity of antibiotics (O R 1.81, CI 1.18–2.78; *P* = 0.002), chronic lung disease (OR 2.02, CI 1.02–3.97; *P* = 0.04), hypoproteinemia (OR 3.59, CI 1.21–10.35; *P* = 0.01), invasive operation before ICU (OR 2.20, CI 1.17–4.11; *P* = 0.01), antibiotics use before ICU (OR 2.95, CI 1.58–5.53; *P* < 0.001), length of hospitalization (OR 1.04, CI 1.02–1.10; *P* < 0.001), length of ICU stay (OR 1.02, CI 1.00–1.05; *P* = 0.04), and long-term bed rest (OR 3.69, CI 1.80–8.12; *P* < 0.001) were risk factors for MDRO infections (Table 3).

**Table 3:** Multivariable logistic analysis for MDRO infection

Predictors	*β*	SE	Wald* χ*^*2*^	OR	95% CI	*P *value
(Intercept)	− 6.81	0.62	− 10.91	0.001	0.000–0.003	< 0.001
APACHE II	0.06	0.02	3.05	1.06	1.02–1.110	0.002
Quantity of antibiotics (categories)	0.59	0.22	2.72	1.81	1.18–2.78	0.002
Chronic lung disease	0.70	0.35	2.03	2.02	1.02–3.97	0.040
Hypoproteinemia, *n* (%)	1.28	0.54	2.37	3.59	1.24–10.35	0.010
Invasive operation before ICU, *n* (%)	0.79	0.32	2.46	2.20	1.17–4.11	0.010
Antibiotics use before ICU, *n* (%)	1.08	0.32	3.40	2.95	1.58–5.53	< 0.001
Length of hospitalization (days)	0.03	0.01	4.23	1.04	1.02–1.05	< 0.001
Length of ICU stay (days)	0.02	0.01	1.98	1.02	1.00–1.05	0.040
Long-term bed rest, *n* (%)	1.30	0.38	3.42	3.69	1.80–8.12	< 0.001

MDRO: multidrug-resistant organism; ICU: intensive care unit; APACHE II: acute physiology and chronic health evaluation II

### Construction and evaluation of the BPNN model

**Fig. 3:**
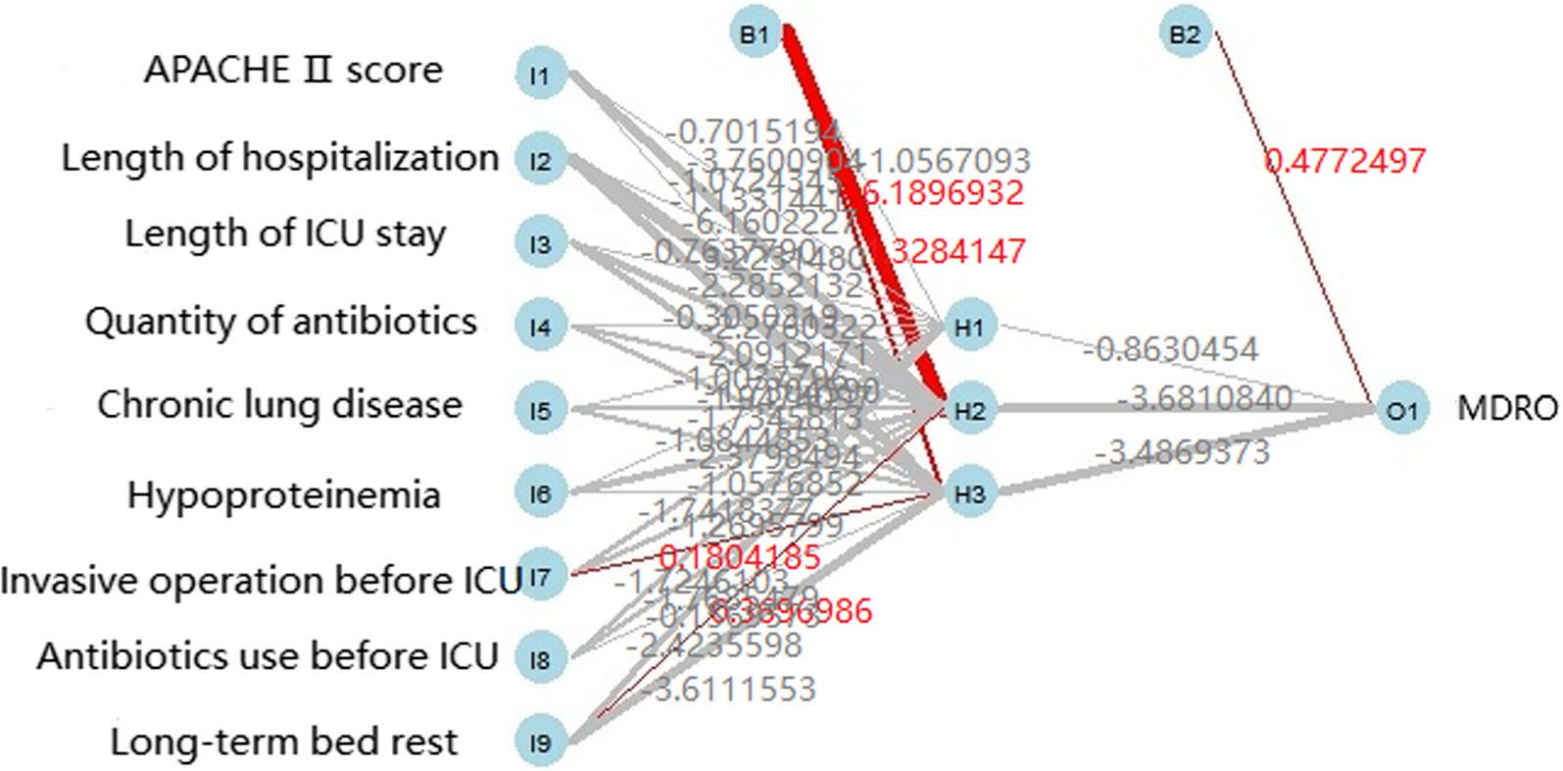
BPNN model for predicting MDRO infection. BPNN: backpropagation neural network; MDRO: multidrug-resistant organism

Nine independent risk factors screened above were employed as input variables to develop the BPNN model (Fig. 3). The parameters of the model were Activation (nonlinear function): logistic, hidden_layer (number of hidden layers): 1, sizes (Number of hidden layer nodes): 3, max_iter (number of iterations): 10, and linout (output function): logistic.

**Fig. 4:**
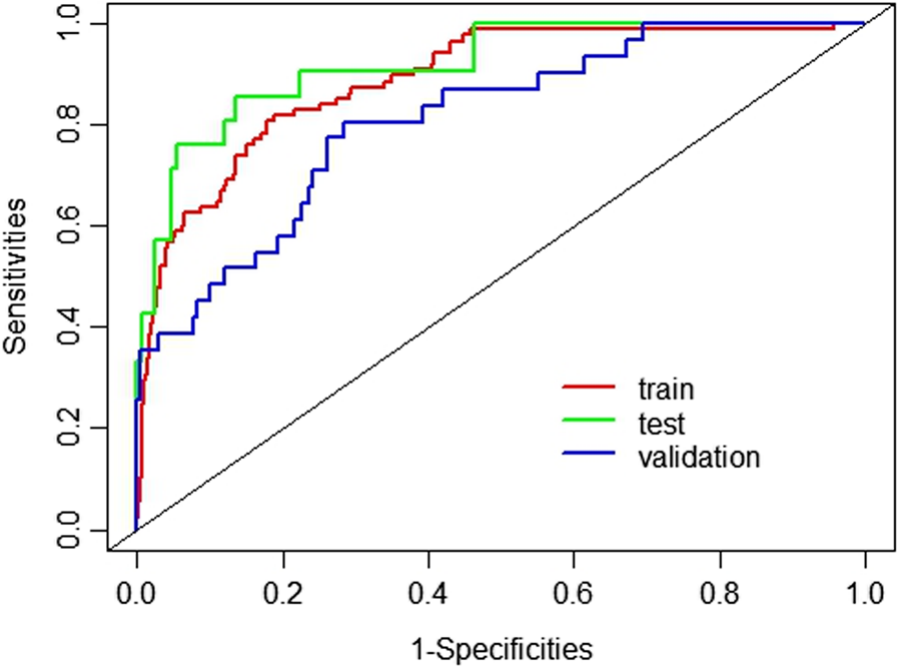
AUCs of the BPNN model of MDRO infection. The *x*-axis represents 1-specifcity, and the *y*-axis represents sensitivity. The part below the red, green and blue lines are the AUCs of the train, testing and validation set. AUC: area under the curve; BPNN: backpropagation neural network; MDRO: multidrug-resistant organism

**Fig. 5:**
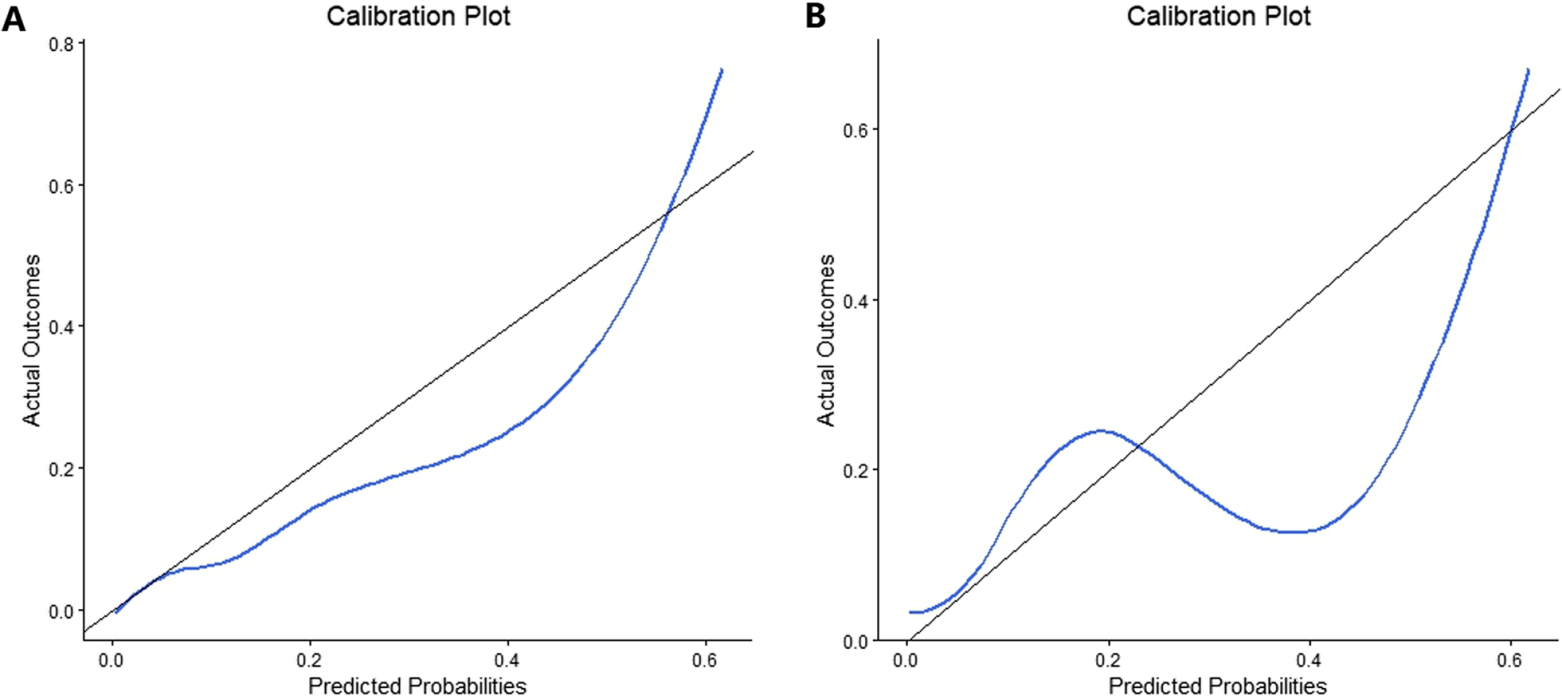
Calibration curves of the BPNN model. The *x*-axis represents the predicted probability of MDRO infection. The *y*-axis represents the actual diagnosed of MDRO infection. The blue solid line represents the perfect prediction with the same predicted probability as the actual probability. The black line represents the performance of the nomogram. The closer the calibration curve of the model is to the black line, the better the model prediction is represented. **A** Calibration curve of the test set. **B** Calibration curve of the validation set. BPNN: backpropagation neural network; MDRO: multidrug-resistant organism

The model's prediction performance was assessed by AUC, accuracy, sensitivity, and specificity, as shown in Table 4. The AUC of the training set and test set were 0.889 and 0.919, respectively. The validation set revealed the same result (AUC = 0.811). Comparisons of the AUC for the model training set, test set, and validation set are depicted in Fig. 4. Calibration curves of the test and validation set showed that the model had good calibration ability (Fig. 5).

**Table 4:** Performance of the BPNN model in the training, test and validation set

Model	AUC (95% CI)	Accuracy	Sensitivity	Specificity
Training set	0.889 (0.852–0.925)	0.895	0.818	0.811
Test set	0.919 (0.856–0.983)	0.918	0.857	0.864
Validation set	0.811 (0.731–0.891)	0.852	0.806	0.715

BPNN: backpropagation neural network; AUC: area under the curve

### Features importance ranking in the BPNN model

**Fig. 6:**
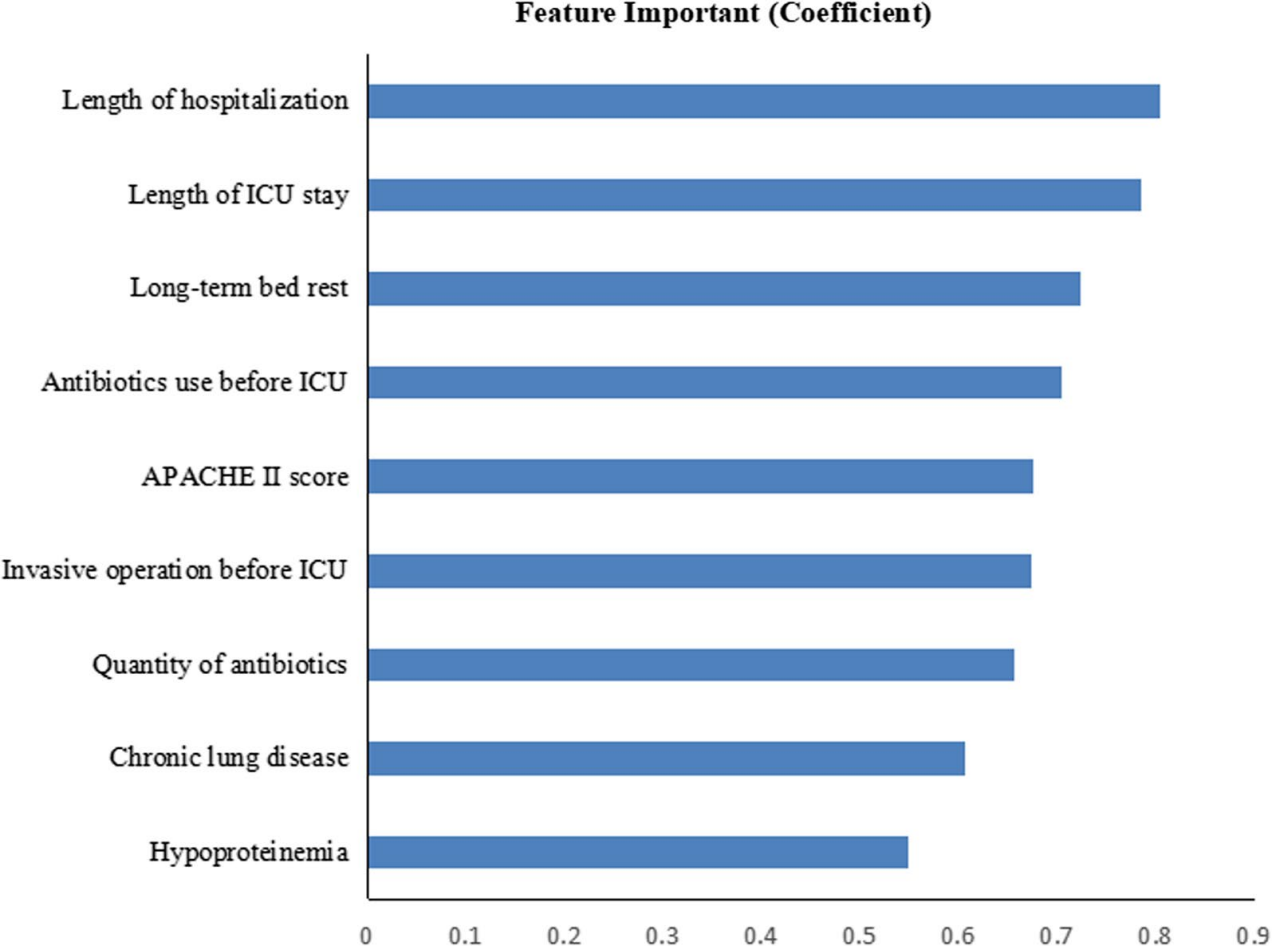
Ranking of features importance in the BPNN model. BPNN: backpropagation neural network

In the BPNN model, the top 5 risk factors affecting MDRO infection were the length of hospitalization, the length of ICU stay, long-term bed rest, antibiotics use before ICU, and APACHE II (Fig. 6).

## Discussion

In this study, we developed and validated an MDRO infection prediction model for ICU patients based on the BPNN algorithm. The model included nine scientifically and clinically accessible independent risk factors: length of hospitalization, length of ICU stay, long-term bed rest, antibiotics use before ICU, APACHE II, invasive operation before ICU, quantity of antibiotics, chronic lung disease, and hypoproteinemia. Utilizing a handful of variables, the BPNN model achieved good performance with high accuracy and sensitivity for predicting the incidence of MDRO infection in ICU patients. Furthermore, we found that the drug-resistant bacteria causing infection in ICU patients were mainly Gram-negative bacteria, especially CR-AB. CR-AB can survive for several days on dry surfaces, and can also be asymptomatic to colonize the skin, respiratory tract, and intestines. Therefore, active monitoring of CR-AB should be strengthened for the high-risk population of MDRO infection.

The prediction model can forecast the risk of individual MDRO infection based on predictors, providing theoretical support for the early identification of high-risk groups and better guidance for formulating MDRO infection management strategies [23, 24]. More and more scholars have begun to explore the construction of the MDRO infection prediction model. Wang et al. collected the data from 331 patients, adopting the method of univariate analysis followed by multivariate analysis. Finally, three risk factors were integrated to build an MDRO infection prediction model with an AUC of 0.77 (95% CI 0.70–0.84) [11]. However, the model's poor performance in predicting MDRO infection risk may be due to other valuable independent variables ignored during the data analysis. The relationships between variables in the ICU are complex, including linear or nonlinear relationships. Nevertheless, LR was used by default to deal with linear relationships between independent and dependent variables and may oversimplify complex nonlinear relationships. BPNN was widely applied in the medical field with its unique advantages, including disease diagnosis, disease classification, prognosis prediction, etc. In this study, the MDRO infection prediction model was constructed using BPNN. The AUC of the training and test sets were 0.889 and 0.918, respectively. Compared with the previous MDRO infection models [25–27], the prediction performance of our BPNN model constructed was improved. In addition, we collected 238 ICU patients' data for external verification. The AUC, accuracy, sensitivity, and specificity were 0.811, 0.852, 0.806, and 0.715, respectively. These results demonstrated that the BPNN model had good discrimination. That suggested that our model had good external applicability and could be used clinically for early prediction of MDRO infection in ICU patients.

In the BPNN model, length of hospitalization, length of ICU stay, long-term bed rest, antibiotics use before ICU, and APACHE II score were the top 5 predictors of MDRO infection. Length of hospitalization and ICU stay were correlated with MDRO infection in ICU patients, which agreed with the conclusions of previous studies [28]. Compared with the non-ICU environment, there are more bacterial isolates in the ICU environment, and the susceptibility is generally lower. ICU patients are more likely to be directly or indirectly exposed to MDRO [29]. As previous evidence indicated, this study also found that long-term bed rest was an independent risk factor for MDRO infection [30]. A meta-analysis showed that prior use of antibiotics, especially third-generation cephalosporin antibiotics, was higher in the multidrug-resistant Gram-negative infection group than in the non-infected group, significantly increasing Gram-negative resistance [31]. This study showed the same results: antibiotics use before ICU was an independent risk factor for MDRO infection. APACHE II score is a tool for evaluating the severity of patients' disease and predicting prognosis. The previous study found that the higher the APACHE II score, the greater the likelihood of MDRO infection and mortality [32]. This study similarly found that the APACHE II score was positively associated with MDRO infection. The previous study showed the association between MDRO and major surgery operation before admission to ICU [33], quantity of antibiotics [34], chronic lung disease [35] and hypoproteinemia [36].

This study has the following advantages. We combined Lasso and stepwise regression to screen for risk factors to avoid multiple collinearity and overfitting of variables. In addition, compared with LR, the BPNN algorithm had strong fault tolerance, nonlinear mapping ability, self-learning and adaptive ability, and generalization ability. Thus, BPNN was employed to mine data characteristics and develop our study's MDRO infection model for ICU patients.

However, it was undeniable that our study had some drawbacks. First, the current study was a single-center retrospective modeling study, which restricted us from determining causal relationships between predictors and outcomes. Therefore, further prospective clinical trials are needed to verify the validity of our model. Second, the retrospective and observational data may result in selection bias. Finally, although external validation was performed in this study, it was limited to data from the same center. In subsequent studies, the sample size can be further expanded, and multicenter studies can be added to optimize the structure of the BPNN model.

## Conclusion

We combined Lasso and backward stepwise regression to screen out nine predictors and built the BPNN model for MDRO infection in ICU patients based on them. The model has proven good prediction performance, which may be an effective instrument for identifying high-risk groups of MDRO infection in the early stage and helping medical personnel intervene early to reduce the rate of MDRO infection in critically ill patients.

### Supplementary Information

**Additional file 1: Table S1**. Frequency of isolated MDRO species. **Table S2.** Comparison of patients’ demographic and clinical characteristics in the primary cohort and validation set.

## Data Availability

The data that support the findings of this study are available on request from the corresponding author upon reasonable request.

## Abbreviations

APACHE II: Acute physiology and chronic health evaluation II
AUC: Area under the curve
BPNN: Backpropagation neural network
CR-AB: Carbapenem-resistant Acinetobacter baumannii
ICU: Intensive care unit
IQR: Interquartile range
Lasso: Least absolute shrinkage and selection operator
LR: Logistic regression
MDRO: Multidrug-resistant organism
NRS: Nutrition risk screening
