# Supplementary material for: A deep learning model for predicting multidrug-resistant organism infection in critically ill patients
Source: J Intensive Care. 2023 Nov 9;11:49. doi: 10.1186/s40560-023-00695-y (PMC10633993; doi:10.1186/s40560-023-00695-y)
Supplement: Supplementary file 1 — Additional file 1: Table S1. Frequency of isolated MDRO species. Table S2. Comparison of patients’ demographic and clinical characteristics in the primary cohort and validation set. [file 40560_2023_695_MOESM1_ESM.doc]

**Table S1** Frequency of isolated MDRO species

| **Isolated MDRO species** | **N=109(%)** |
| --- | --- |
| **Carbapenem resistant Acinetobacter baumannii** | 28(25.69) |
| **Carbapenem resistant Pseudomonas aeruginosa** | 19(17.43) |
| **Extended-spectrum β-lactamase-producing Klebsiella pneumoniae** | 16(14.68) |
| **Extended-spectrum β-lactamase-producing Escherichia coli** | 13(11.93) |
| **Meticillin-resistant Staphylococcus aureus** | 10(9.17) |
| **Meticillin-resistant** **Coagulase-negative staphylococci** | 9(8.26) |
| **Carbapenem resistant Escherichia coli** | 4(3.67) |
| **Meticillin-resistant Coagulase Staphylococcus epidermidis** | 3(2.75) |
| **Carbapenem-resistant Klebsiella pneumoniae** | 3(2.75) |
| **high-level aminoglycoside resistant enterococcus** | 2(1.83) |
| **Vancomycin-resistant enterococci** | 1(0.92) |
| **Carbapenem-resistant Enterobacter cloacae** | 1(0.92) |

Abbreviations MDRO: Multidrug-resistant organisms

**Table S2** Comparision of patients’ demographic and clinical characteristics in the primary cohort and validation set

| **Variables** | **Total (n =926)** | **Primary cohort (n =688 )** | **Validation set (n =238 )** | ***P* value** |
| --- | --- | --- | --- | --- |
| **Length of hospitalization(days)** | 18.00 (11.00 – 29.00) | 19.00 (11.00 – 29.00) | 18.00 (10.00 – 29.00) | 0.364 |
| **Length of ICU stay(days)** | 9.00 (5.00 – 15.00) | 9.00 (5.00 – 16.00) | 8.00 (5.00 – 14.00) | 0.115 |
| **APACHE Ⅱ score** | 16.00 (12.00 – 21.00) | 17.00 (12.00 – 21.00) | 15.00 (11.00 – 21.00) | 0.036 |
| **Quantity of antibiotics (categories)** | 1.00 (1.00 – 1.00) | 1.00 (1.00 – 1.00) | 1.00 (1.00 – 1.00) | 0.215 |
| **Long-term bed rest, n (%)** | 423 (45.70) | 361 (52.50) | 62 (26.10) | <0.001 |
| **Antibiotics use before ICU, n (%)** | 277 (29.90) | 212 (30.80) | 65 (27.30) | 0.309 |
| **Invasive operation before ICU, n (%)** | 177 (19.10) | 141 (20.50) | 36 (15.10) | 0.069 |
| **Chronic lung disease** | 112 (12.10) | 85 (12.40) | 27 (11.30) | 0.070 |
| **Hypoproteinemia, n (%)** | 36 (3.90) | 26 (3.80) | 10 (4.20) | 0.680 |
| **MDRO, n (%)** | 140 (15.10) | 109 (15.80) | 31(13.00) | 0.296 |

Abbreviations APACHE Ⅱ: Acute physiology and chronic health evaluation Ⅱ; AUC: Area under curve; ICU: Intensive care units; BPNN: Back propagation neural network; Lasso: Least absolute shrinkage and selection operator; LR: Logistic regression; MDRO: Multidrug-resistant organisms; IQR: interquartile range.
